# Supplementary material for: Variant PCGF1-PRC1 links PRC2 recruitment with differentiation-associated transcriptional inactivation at target genes
Source: Nat Commun. 2021 Sep 9;12:5341. doi: 10.1038/s41467-021-24894-z (PMC8429492; doi:10.1038/s41467-021-24894-z)
Supplement: Supplementary file 1 — Supplementary Information [file 41467_2021_24894_MOESM1_ESM.pdf]

Supplementary Information

Supplementary Figure Legends

Supplementary Figure 1.

The bar graph showing representative Gene Ontology (GO) terms related to "biological process" for each group of genes. The p-value are calculated by Fisher Exact Statistics adjusted by Benjamini-Hochberg correction via DAVID software (v6.7).

Supplementary Figure 2.

a, Gene expression of representative PcG factors during ESC-to-EB differentiation. The bar graph shows mRNA expression (Transcript Per Million: TPM) of the indicated genes. Error bars represent SEM (n=2).

b, The sampling scheme of respective mutant ESCs and EBs.

c, Gene expression changes in each group upon deletion of *Ring1a/b*, *Pcgf1*, *Pcgf2/4*, *Pcgf3/5* or *Pcgf6* in ESCs and EBs. Violin plots show mRNA expression (Transcript Per Million: TPM) of the indicated genes in each group in WT (*fl/fl*) or mutant (Δ/Δ) ESCs and EB. The genes exhibiting more than a 2-fold increase (Log2FC([Δ/Δ]/[*fl/fl*])>1) or decrease (Log2FC([Δ/Δ]/[*fl/fl*])<1) are connected by red or blue lines, respectively.

d, Average binding of RING1B in each gene group in WT or *Pcgf6*-KO ESCs and EBs. Metaplots of RING1B ChIP-Seq at TSS ± 5kb of each gene group in WT (*fl/fl*) or *Pcgf6*-KO (Δ/Δ) ESCs and EBs are shown. Data for WT or *Pcgf6*-KO are shown in solid and dotted lines, respectively. Data from ESCs or EBs are shown in green and pink lines, respectively.

e. Genomic snapshots showing RING1B distributions in WT (fl/fl) and *Pcgf6*-KO (Δ/Δ) ESCs and EBs. Gene structures and the position of CpG islands are indicated at the bottom.

f. The sampling scheme for WT (*fl/fl*) or *Pcgf1*-KO (Δ/Δ) EpiLC.

g. Representative morphology of the WT (*fl/fl*) or *Pcgf1*-KO (Δ/Δ) EpiLCs. WT (*fl/fl*) or *Pcgf1*-KO (Δ/Δ) EpiLCs exhibited similar cellular morphology. Experiment were repeated independently at least 2 times with same results.

h. Accumulation of RING1B and H3K27me3 at *Klf4*, *Tbx3* genes in WT (*fl/fl*) or *Pcgf1*-KO (Δ/Δ) ESCs and EpiLCs. Bar graphs for ChIP-qPCR results for RING1B and

H3K27me3 in WT (*Pcgf1*$^{fl/fl}$) or *Pcgf1*-KO (*Pcgf1*$^{\Delta/\Delta}$) ESCs and EpiLCs are shown. Error bars represent SEM (n=2).

i, Progressive expression changes of selected Group1 genes (*Klf4*, *Tbx3*, *Pdgfa*) and Group3 genes (*Sox4*, *Grhl2*) in WT (*fl/fl*) or *Pcgf1*-KO (Δ/Δ) ESCs during ESC-to-EB differentiation. The line plots show RT-qPCR results during ESC-to-EB differentiation (at 0 hrs, 4 hrs, 8 hrs, 24 hrs, and 48 hrs) in WT (fl/fl) or *Pcgf1*-KO (Δ/Δ) ESC. Y-axis is shown by logarithmic scale. *p*-value shows the significant differences calculated by Student's t test (n=6).

j, Progressive RING1B, SUZ12 and PCGF2 binding changes at selected Group1 genes (*Klf4*, *Tbx3*, *Pdgfa*) and Group3 genes (*Sox4*, *Grhl2*) during ESC-to-EB differentiation. Error bars represent SEM (n=2).

k, The expression of selected Group1 (*Klf4*, *Tbx3*) and Group3 (*Sox4*, *Grhl2*) genes in WT (fl/fl) or *Pcgf1*-KO (Δ/Δ) epiSCs. Bar graphs for their expression revealed by RT-qPCR in WT (fl/fl) or *Pcgf1*-KO (Δ/Δ) epiSCs are shown. *Pou5f1* was used as a control. Error bars represent SEM (n=2).

l, Generation and colony morphology of WT (fl/fl) or *Pcgf1*-KO (Δ/Δ) epiSCs. WT (fl/fl) or *Pcgf1*-KO (Δ/Δ) epiSCs exhibited aimilar colony morphology. Experiment were repeated independently at least 2 times with same results.

Supplementary Figure 3.

a, Distribution of H2AK119ub1 in each gene group in WT (fl/fl) or *Pcgf1*-KO (Δ/Δ) ESCs and EB. Metaplots of H2AK119ub1 revealed by CUT&Tag analysis at TSS ± 5kb of each gene group in WT (fl/fl) or *Pcgf1*-KO (Δ/Δ) ESCs and EB are shown. Data for WT (fl/fl) or *Pcgf1*-KO (Δ/Δ) are shown in solid and dotted lines, respectively. Data for ESCs and EBs are shown in green and pink lines, respectively.

b, Gene expression changes in each group in WT or *Ring1A/B* point mutant ESCs and EB. 4-OHT was added to WT (*Ring1a*$^{pm/pm}$*::Ring1B*$^{cpm/cpm}$*::ERT2-Cre,* indicated as -4OHT) to obtain *Ring1A/B* point mutants. Violin plot shows mRNA expression (TPM: Transcript Per Million) of genes in each group in WT (-4OHT) or RING1 point mutant (+4OHT) ESCs and EB. genes exhibiting more > 2-fold increase (Log2FC([Δ/Δ]/[*fl/fl*])>1) or decrease (Log2FC([Δ/Δ]/[*fl/fl*])<1) are connected by red and blue lines, respectively.

c, Schematic representation of the targeting vector to generate the *Eed* conditional allele. loxP sites (open triangles), FRT sites (bold arrows), Neomycin-resistant gene cassette (Neo) and exons (closed bars) are indicated. The neomycin-resistant gene cassette was deleted by expressing FLP-recombinase in homologous recombinant ESCs. PCR primers used for genotype analysis are indicated by arrows.

d, Schematic representation for WT or mutant (ΔExon3) EED. ΔExon3 version of EED lacks the first WD40 motif.

e, Deletion of exon 3 by 4-OHT treatment in $Eed^{fl/fl}$*::Rosa26*$^{CreERT2tg/+}$ ESCs revealed by genomic PCR analysis. Experiment were repeated independently 3 times with same results.

f, Truncation of EED protein, and depletion of H3K27me3, by 4-OHT treatment in $Eed^{fl/fl}$*::Rosa26*$^{CreERT2tg/+}$ ESCs detected by WB analysis. The blot of EED and H3K27me3 were derived from the same blot membrane. After transfer, the membrane was cut by expected molecular size and reaction the antibody individually. The blot of b-actin was reacting with antibody to same blot membrane of EED which were stripped the antibody. And the biological replicate was done two times with the same results.

g, Genomic snapshots of PCGF1 distributions in WT (*fl/fl*) or *Eed*-KO (Δ/Δ) ESCs and EBs are shown. Gene structures and position of the CpG islands (CGI) are indicated at the bottom.

Supplementary Figure 4.

a. Violin plots showing RNAPII binding at TSS ± 5kb of each gene, with or without, Triptolide treatment. Significant differences were calculated by Mann–Whitney U test (one sided). Data from the control (DMSO) or Triptolide-treated (Triptolite) ESCs are shown in green and purple, respectively.

b. Distribution of RING1B and SUZ12 in each cluster in WT or *Pcgf1*-KO with or without triptolide treatment. Metaplots of RING1B and SUZ12 ChIP-Seq at TSS ± 5kb of the genes in each cluster in WT (*fl/fl*) and *Pcgf1*-KO (Δ/Δ) ESCs with or without triptolide treatment are shown. Data from the control (DM) or Triptolide-treated (TR) ESCs are shown in green and purple lines, respectively.

c. Heatmap views showing changes in SUZ12 distribution around TSS (± 5kb) of each gene as revealed by a previous study[3] (SRR1300952 [DMSO treated ESC] [https://www.ncbi.nlm.nih.gov/sra/?term=SRR1300952] and SRR1300956 [Triptolide treated ESC] [https://www.ncbi.nlm.nih.gov/sra/?term=SRR1300956]). Genes were clustered as shown in Figure 4a.

d. Venn diagrams showing the overlaps between Clusters 3 and 4 with Group 1 genes (defined in Figure 1b). The number of genes in each fraction is indicated. Names of genes in each overlap are shown. Note *Klf4*, *Tbx3* and *Pdgfa* are included in Cluster 4.

Supplementary Figure 5.

a, Dox-dependent regulation of H3K27 acetylation at the reporter gene in ESCs. Snapshot views at the reporter gene revealed by CUT&Tag analysis for H3K27 acetylation in WT (*fl/fl*) or *Pcgf1*-KO (Δ/Δ) ESCs are shown. Positions of CGI, TRE3G, and *EGFP* regions are shown below. H3K27 acetylation at *Gapdh* and *Meis2* are shown as reference values.

b, Dox-independent regulation of KDM2B binding at the CGI region of the reporter gene. Bar graphs show ChIP-qPCR results of KDM2B accumulation at human CGI on the reporter gene, *Gapdh*, *Meis2*, and *Nanog* are controls that do not possess CGI in ESCs (H3K27me3 and H2AK119ub1: n=2, RING1B, SUZ12 and MEL18: n=3).

c, Dox-dependent accumulation of PCGF1 at the CGI region of the reporter gene. Bar graphs show ChIP-qPCR results of PCGF1 at human CGI on the reporter gene, *Gapdh* and *Meis2* also shown as CGI+ examples, and Nanog as a CGI- example in *Pcgf1*-KO ($Pcgf1^{\Delta/\Delta}$) ESCs and those expressing exogenous PCGF1-TY1 ($Pcgf1^{\Delta/\Delta}$-PCGF1TY1). Error bars represent SEM (n=3).

d, Dox- and PCGF1-dependent regulation of the distribution of H2AK119ub1 and H3K27me3 at the reporter gene in ESCs. Snapshot views at the reporter gene revealed by CUT&Tag analysis for H2AK119ub1 and H3K27me3 in WT (*fl/fl*) or *Pcgf1*-KO (Δ/Δ) ESCs are shown. Their distribution at *Gapdh* and *Meis2* are shown as reference values.

e, Morphological and growth defects in *Pcgf1*-KO embryos at 9.5 dpc. Lateral views of WT (fl/fl) or *Pcgf1*-KO (Δ/Δ) reveal delayed elongation and outgrowth. This phenotype was observed independently in 5 embryos with same results.

Supplementary Figure 1

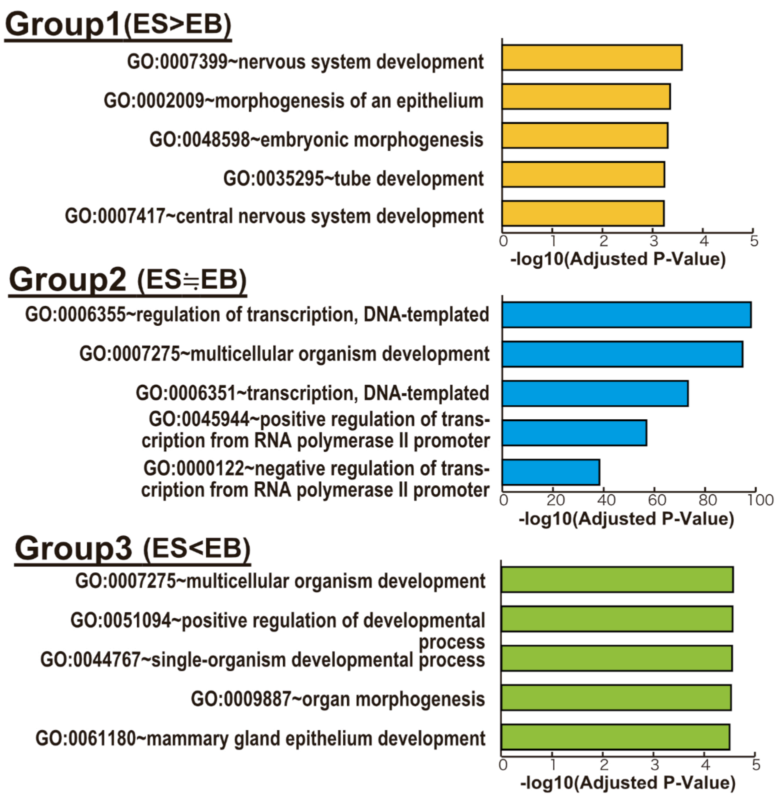

Supplementary Figure 2

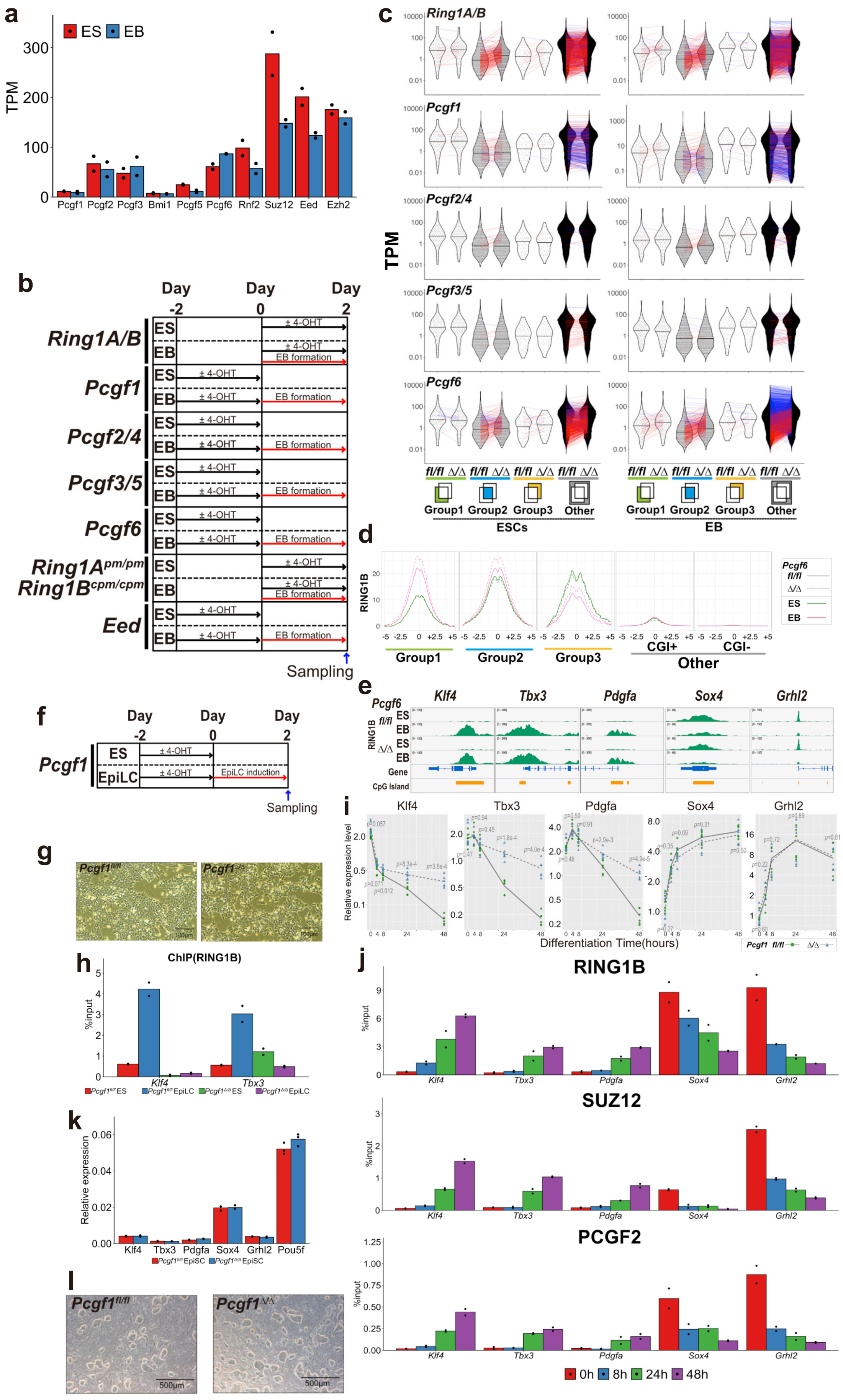

Supplementary Figure 3

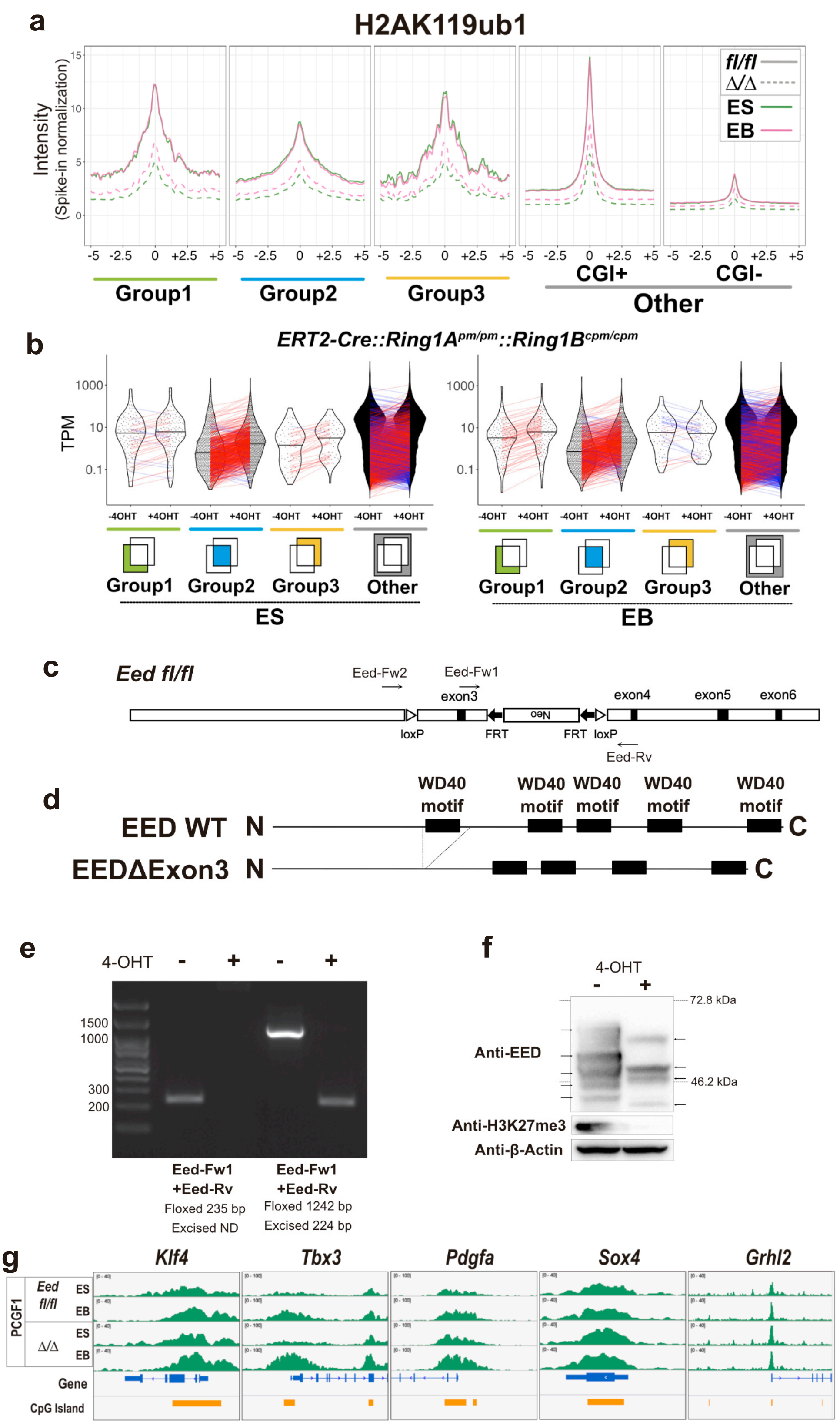

Supplementary Figure 4

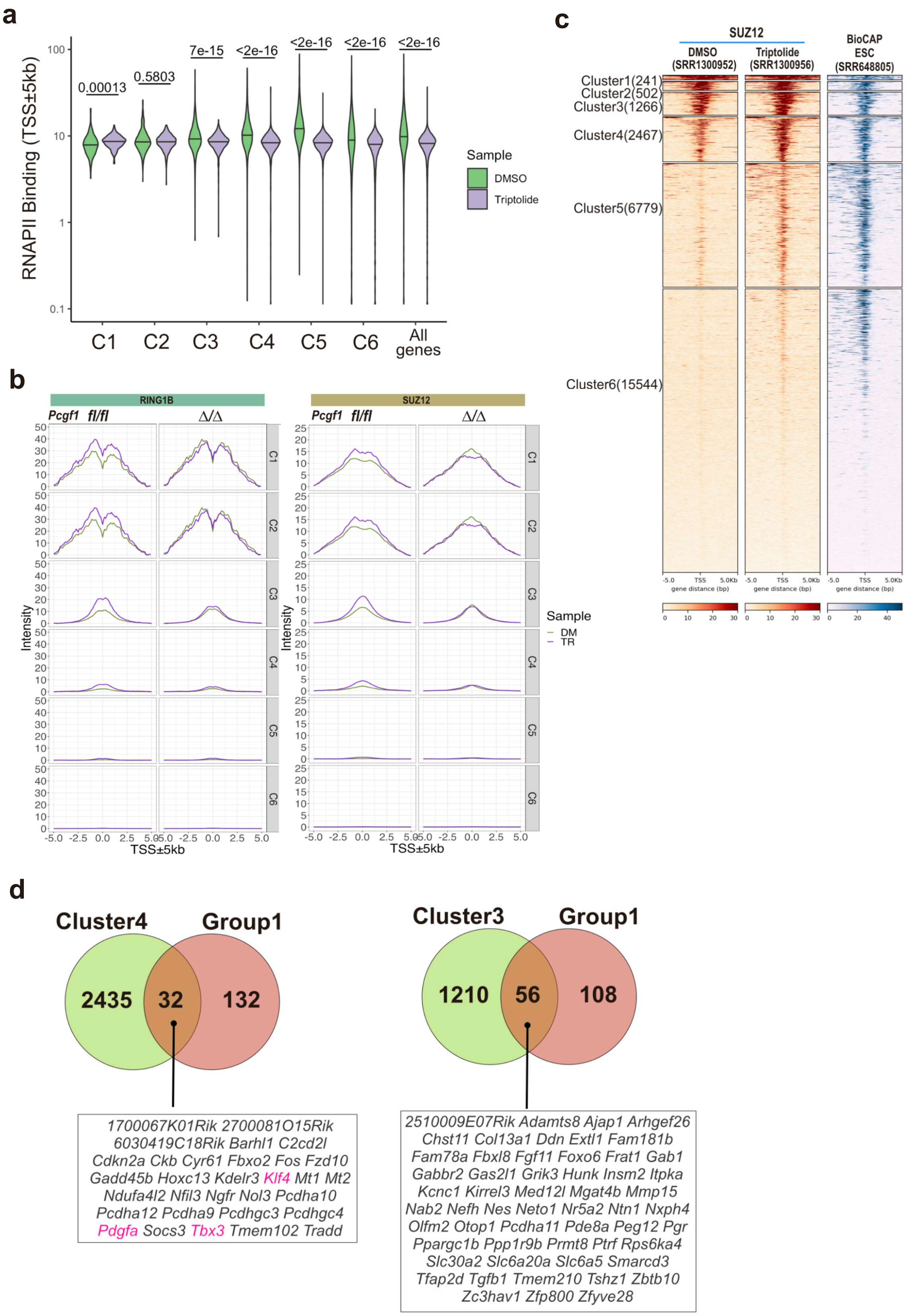

Supplementary Figure 5

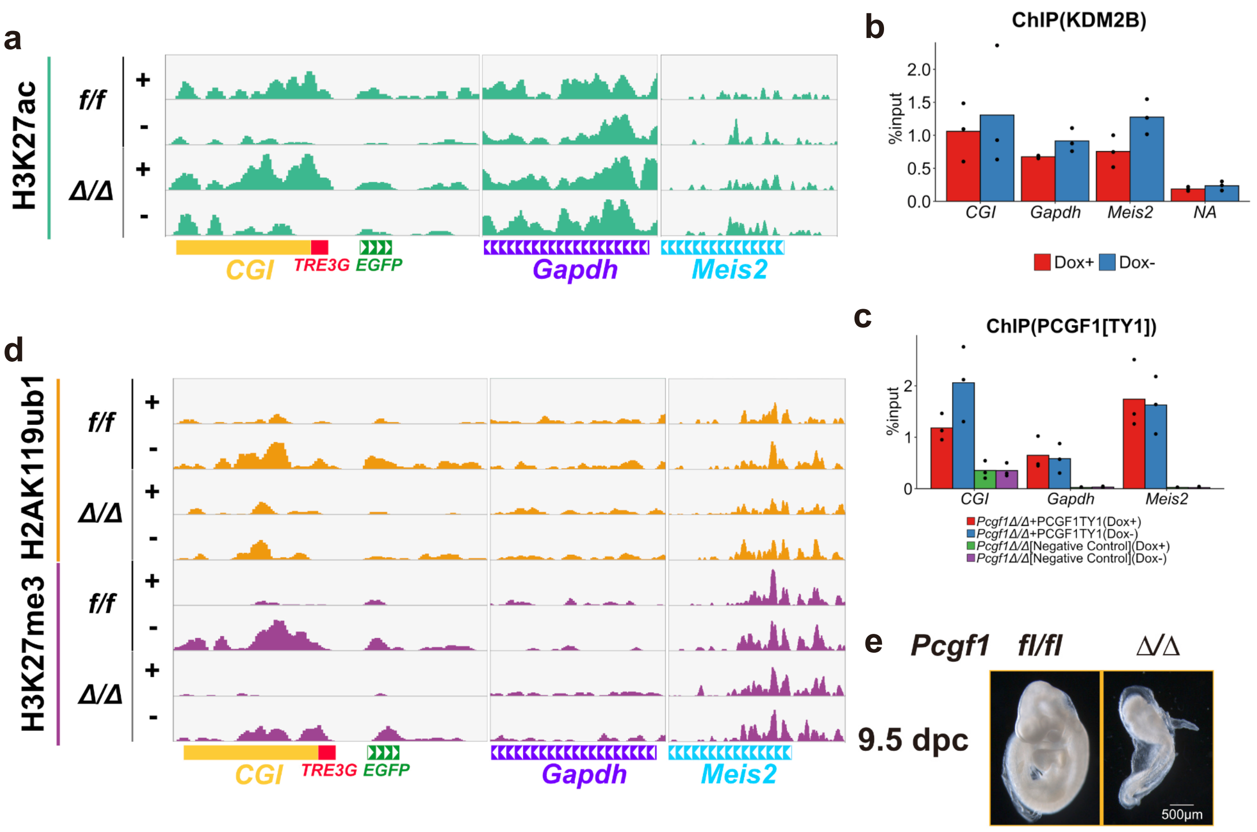

Application	Name	Sequence
ChIP-qPCR	Dox_CGI_Fw	TCCCATCTTTCTCCACGTTC
ChIP-qPCR	Dox_CGI_Rv	TCCTTACCCTCGTTCAGTGG
ChIP-qPCR	Klf4_Fw	GGACCTACTTATCTGCCTTGC
ChIP-qPCR	Klf4_Rv	GAGCCCCAAAGTCAACGAAG
ChIP-qPCR	Tbx3_Fw	GAATTCTAGAGGCGGAGGAG
ChIP-qPCR	Tbx3_Rv	AGTGATTGGGAGCTGGAGTAG
ChIP-qPCR	Pdgfa_Fw	CCCCCGAAGTTTCTACCCAC
ChIP-qPCR	Pdgfa_Rv	TGGTTTTGTCAGGGTGGTGT
ChIP-qPCR	Grhl2_Fw	TTCTTTGCTTCCTCCCCGAC
ChIP-qPCR	Grhl2_Rv	TCGACTGAACTTCCAACCGG
ChIP-qPCR	Sox4_Fw	GCAAGATCATGGAGCAGTCG
ChIP-qPCR	Sox4_Rv	CCTGGATGAACGGAATCTTG
ChIP-qPCR	Meis2_Fw	GATGAGTGAGTGTCAGTAGG
ChIP-qPCR	Meis2_Rv	GGTTCCGTCATTTCGTTCTC
ChIP-qPCR	Nanog_Fw	TAGGGTAGGAGGCTTGAGGGGGGA
ChIP-qPCR	Nanog_Rv	CAGCCTTCCCACAGAAAGAGCAAGACAC
ChIP-qPCR	Gapdh_Fw	CTTCGGGCCACGCTAATCTCATTT
ChIP-qPCR	Gapdh_rv	AACTCACCCGTTCACACCGACCTT
RT-qPCR	Pdgfa_qRT_Fw	CTCTTGGAGATAGACTCCGTAGG
RT-qPCR	Pdgfa_qRT_Rv	ACTTCTCTTCCTGCGAATGG
RT-qPCR	Grhl2_qRT_Fw	TTTGGTCCAACACCGTCTA
RT-qPCR	Grhl2_qRT_Rv	CACTGGCAGCCCATACTT
RT-qPCR	Klf4_qRT-Fw	CGGGAAGGGAGAAGACACT
RT-qPCR	Klf4_qRT-Rv	GAGTTCCTCACGCCAACG
RT-qPCR	Tbx3_qRT-Fw	GAACCTACCTGTTCCCGGAAA
RT-qPCR	Tbx3_qRT-Rv	CCATTGCCAGTGTCTCGAAAAC
RT-qPCR	Sox4_qRT_Fw	GCAAGATCATGGAGCAGTCG
RT-qPCR	Sox4_qRT_Rv	CCTGGATGAACGGAATCTTG
RT-qPCR	Gapdh_qRT_Fw	GACATGCCGCCTGGAGAAAC
RT-qPCR	Gapdh_qRT_rv	AGCCCAGGATGCCCTTTAGT
PCGF1-TY1 constructioon	3TY1-mPcgf1-1st-Fw	CTGGATGCCGAAGTGCACACCAATCAGGATCCCCTGGACGCTGGGATCCAGAGGCTTCGGAACCAGCTCC
PCGF1-TY1 constructioon	3TY1-2nd-Fw	ACACCAACCAGGACCCCCTGGACGCCGAAGTCCATACAAATCAGGATCCTCTGGATGCCGAAGTGCACAC
PCGF1-TY1 constructioon	XhoI-3TY1-3rd-Fw	CTCACTCGAGATGGAGGTGCACACCAACCAG
PCGF1-TY1 constructioon	mPcgf1-NotI-Rv	CTATGCGGCCGCCTACCTCCTCTTCTCTTTCACAC
CGI activity detection system	hKLF4_CGI_Fw	TATGCTCTCGTTAATCCTCCTCTCCACACCCCTAG
CGI activity detection system	hKLF4_CGI_Rv	ATAGGGAGTAAATTATAGTCCTGCTGGGGAGCTG

Supplementary Table 1 |Primer list

Name	Application	Host	Supplier
Anti-RING1B	ChIP-qPCR(1:20)/ChIP-Seq(1:20)/Western Blotting(1:200)	Mouse	Laboratory made(Yakushiji-Kaminatsui, N., et al. (2016). Development 143, 276-285; Bravo, M., et al. (2015). J. Cell Sci. 128; 3660-3671; Atsuta, T., et al. (2001). Hybridoma. 20(1); 43-
Anti-MEL18	Anti-MEL18 ChIP-qPCR(1:20)	Rabbit	Laboratory made,We validated the antibody's specificity in this study(see Source file)
Anti-KDM2B	ChIP-qPCR(1:20)/ChIP-Seq(1:20)/Western Blotting(1:200)	Mouse	Laboratory made, We validated the antibody's specificity in this study(see Source file)
Anti-SUZ12	ChIP-qPCR(1:50)	Rabbit	CST (#3737S)
Anti-H2AK119ub	ChIP-qPCR(1:50)	Mouse	Sigma(05-678)
Anti-PCGF1	ChIP-qPCR(1:50)/ChIP-Seq(1:50)	Rabbit	Laboratory made(Blackledge et al., 2014, Cell; Fursova et al., 2019, Molecular Cell)
Anti-PCGF1	Western Blotting(1:200)	Mouse	Santa Cruz(SC515371)
Anti-TY1	ChIP-qPCR(1:50)	Mouse	Sigma(SAB4800032-50UG)
Anti-H3K27me3	ChIP-qPCR(1:50),Cut&Tag(1:100)	Rabbit	CST(#9733S)
Anti-H2AK119ub	Cut&Tag(1:100)	Rabbit	CST (#8240S)
Anti-H3K27ac	Cut&Tag(1:100)	Rabbit	CST (#8173S)

Supplementary Table 2 |Antibody list
