# Supplementary material for: Variant PCGF1-PRC1 links PRC2 recruitment with differentiation-associated transcriptional inactivation at target genes
Source: Nat Commun. 2021 Sep 9;12:5341. doi: 10.1038/s41467-021-24894-z (PMC8429492; doi:10.1038/s41467-021-24894-z)
Supplement: Supplementary file 3 — Description of Additional Supplementary Files [file 41467_2021_24894_MOESM3_ESM.docx]

**Description of Additional Supplementary Files**

File name: Supplementary Data 1

Description: Gene lists of Group 1, 2 and 3 genes shown in Figure 1b.

File name: Supplementary Data 2

Description: Fold change score of gene expression upon ESC-to-EB differentiation.

File name: Supplementary Data 3

Description: Gene lists of Cluster 1, 2, 3, 4, 5 and 6 genes shown in Figure 4a.
